# Mentoring in the clinical training of midwifery students - a focus study of the experiences and opinions of midwifery students at the Medical University of Warsaw participating in a mentoring program

**DOI:** 10.1186/s12909-020-02324-w

**Published:** 2020-10-30

**Authors:** Małgorzata Stefaniak, Ewa Dmoch-Gajzlerska

**Affiliations:** Department of Obstetrics and Gynecology Didactics, Faculty of Health Sciences, Medical University of Warsaw, Litewska Str. 14/16, 00-575 Warsaw, Poland

**Keywords:** Mentoring, Midwifery, Undergraduate students, Education, Undergraduate clinical training

## Abstract

**Background:**

The current system of clinical training for midwifery students in Poland is in need of considerable revision to adapt it to the global standards and the expectations of healthcare providers, healthcare users and student midwives themselves. Aim of this study was to report the experiences of midwifery students participating in a mentor-led clinical training program and their opinions of mentoring as a novel training method.

**Methods:**

A qualitative descriptive study that used a focus group was undertaken in the period from October 2017 to June 2019. The participants were 12 s- and third-year midwifery students at the Medical University of Warsaw who at various times during the study period had their clinical training in the Department of Obstetrics, Solec Hospital in Warsaw, Poland. All students had previous experience of clinical training other than clinical mentorship. At the end of the study, a focus group interview was conducted with all 12 participants. Five questions were selected to guide the focus group discussion: *Did you get any valuable learning experience during your clinical training? How did this clinical training differ from your previous clinical training? What was your experience of one-on-one mentoring? Did the mentoring program meet your expectations? What do you think could be changed to make the proposed mentor-led clinical training more effective?*

**Results:**

Four themes were identified. The study demonstrated that mentoring was perceived by the participants as an innovative and effective method of clinical training for midwifery students. All students positively evaluated the quality of the mentor-led clinical training which allowed improving their clinical skills and building new competencies. Students believed they could effectively use their clinical skills and make informed decisions in a safe and supportive clinical learning environment. They felt that their inclusion in the therapeutic team contributed to better patient care.

**Conclusions:**

The use of innovative forms of clinical training at undergraduate level improves its effectiveness and in the future should be reflected in a high-quality maternity care. Mentoring has its advantages for both, mentor and mentee, but the main goal is to develop and improve professional competencies of the junior partner.

**Supplementary Information:**

The online version contains supplementary material available at 10.1186/s12909-020-02324-w.

## Background

High-quality maternity care is a major goal of the state health policy in Poland as seen in the *Programme for complex protection of reproductive health in Poland 2016–2020* [1]. The mandatory introduction of standards and procedures for perinatal care described in the *Regulation of Minister of Health of 16 August 2018 on the organizational standards of perinatal care* is considered to be of key importance for the unification of standards of routine perinatal care and management of complications [2]. It also contributes to increasing the competencies of midwives who are required to assume responsibility for own decisions and actions as autonomous health care practitioners [3].

Midwifery educators are expected to design curricula and teaching/ learning models that would prepare newly qualified midwives to deliver high-quality, evidence-based maternity care meeting the updated standards [4]. In accordance with the European Union requirements, “the training must be given on a full time basis and comprise at least 3 years or 4 600 hours; clinical training must constitute one half of the training” [5]*.* This shows that clinical skills are to be gained from work experience while hands-on training is seen as a crucial component of undergraduate midwifery courses.

Medical universities offering midwifery degree courses are increasingly challenged to update the curricula and seek novel approaches to clinical training [6]. It is expected that innovative methods of undergraduate clinical training will contribute to a better quality of patient care in the future [7, 8]. The use of mentoring to midwifery students is regarded as a key new addition to traditional clinical placements [9–11].

Mentoring was first used in the training of nursing students and junior nurses at The Flinders University of South Australia (FUSA) School of Nursing [12]. The aim was to help mentees in developing competencies, increasing their confidence, building partnerships and facilitating professional development. Nowadays, mentoring as a method of training and providing support to nurses has been adopted globally and in Poland it was first implemented at the Jagiellonian University Medical College in Cracow in collaboration with Sheffield Hallam University. Educators from these two institutions developed an innovative program preparing nurses to act as mentors, including a set of guidelines for mentorship in nursing [13].

In Poland, clinical training for midwifery students is conducted in groups usually consisting of 4 to 8 students who are supervised by an academic/ clinical tutor and this concept of clinical training does not sufficiently contribute to effective skills development nor does it satisfy the expectations of many undergraduates. However, a fair number of instructors are wary of educational innovations and do not perceive their benefits for students and healthcare in general while others may actually provide mentoring for midwifery students training in their maternity wards without formally recognizing it as an inherent part of the curriculum [14–17].

Globally, the concept of mentoring in the education and training of nurses has been widely discussed reflecting numerous instances of its actual implementation, but there have been significantly fewer published papers on mentoring for midwives and midwifery students [14–20]. Traditional clinical placements as a method of training nurses and midwives do not seem to satisfy all criteria of efficient and effective new knowledge acquisition [6, 20]. In midwifery as in nursing, the level of professional knowledge and preparedness to deliver clinical care are the outcome of undergraduate clinical training and depend on its quality [4, 7, 21]. When a midwifery student can collaborate with a midwife and become a member of the therapeutic team, this enhances the student’s sense of belonging in the hospital department and helps her/him to learn more effectively, not just mechanically memorize the prescribed procedures and blindly accept the instructions. A better understanding of the problem and knowledge retention are achieved, with focus on the ‘Why’, not just the ‘What’ and the ‘How’ [22–24].

Mentor-led clinical training has been seen by many nursing and midwifery educators as a solution leading to the improved acquisition of clinical skills and developing students’ sense of empowerment [19, 25, 26]. Mentoring is defined as a relationship between a person who is just starting their career (*mentee*) and an experienced practitioner (*mentor*) who provides support, advice and knowledge which the mentee needs for her/his personal and professional development [27, 28]. The relationship is voluntary and involves socialization, development and building trust, and is a way of realizing personal and professional potential. It is beneficial to both, mentor and mentee, but the primary goal is to improve the effectiveness of the junior partner’s performance [17, 29]. The mentoring process assumes a partnership between midwifery mentors and clinicians who have joint responsibility for the clinical training.

Mentoring may improve the quality of clinical training by focusing on the development of competencies and introducing midwifery students to the real-world health care. A safe and supportive clinical learning environment contributes to the effective use of acquired clinical skills and informed decision making [30] while positive reinforcement of the students’ efforts and accomplishments by experienced midwives who act as mentors plays an important role in preparing newly qualified midwives to work as autonomous practitioners [25, 26]. Mentoring makes students more active in the learning process, improves the retention of clinical knowledge, develops clinical skills and shows mentees ways to learn and to manage time effectively.

In the academic year 2017/18, the Medical University of Warsaw was the first medical school in Poland to implement a pilot program of mentor-led clinical training for midwifery students. The aim of the present study was to evaluate the clinical learning experience of midwifery students who had completed a three-week cycle of one-one-one clinical instruction. This is the first paper which reports the study findings, including evaluation by students of individual versus group training. We hope that this paper will to some extent fill a current gap in research and promote adoption of individualized mentoring perceived by students as an innovative and effective form of clinical training.

## Methods

### Pre-study preparation

Prior to the program initiation, the design of the study, concept of mentoring, sequential phases of establishing a mentoring relationship and information on implementing particular stages of the proposed mentor-led clinical training in midwifery were presented in a guide available to candidate participants, both mentors and mentees.

### Setting

The Department of Obstetrics and the Delivery Unit, Solec Hospital in Warsaw, the clinical training site for midwifery students at the Medical University of Warsaw.

### Participants

Twelve second- and third- year midwifery students at the Medical University of Warsaw who had their three-week clinical mentoring program at various times from October 2017 to June 2019. All students had previous experience of traditional clinical training of the same length but conducted in groups of 4 to 8 students.

### Sampling

Mentees were recruited based on their academic performance (a high mean grade achieved in all examinations in the preceding academic year) and declared interest in individual teaching and learning as well as willingness to develop their clinical competencies and skills through mentoring. The recruitment was carried out by the program coordinator supported by a psychologist and was based on an individual interview and a self-assessment questionnaire which included such items as experience of earlier clinical training, motivation to participate in the mentoring program, expectations concerning the program and mentor, perceived personal strengths and weaknesses, clinical skills the student wanted to develop and support they expected.

The mentors were 12 midwives which had at least 2 years’ work experience and were members of the staff at the Department of Obstetrics, Solec Hospital in Warsaw and preferably met such additional requirements as a higher degree in midwifery, teaching qualifications, completed postgraduate courses and experience in clinical training of midwifery students. The prospective mentors had to complete a self-assessment questionnaire which included such items as the length of employment at the Department, position, experience of previous clinical training of student midwives, motivation to act as a mentor and expectations related to the program. They were given professional training in mentor-led clinical instruction which focused on the concept of mentoring, design of the mentoring program, identification of mentees’ needs and setting measurable learning objectives and key competencies, provision of a safe and supportive learning environment, effective methods of mentor-led clinical instruction and principles of student appraisal. Based on a thorough analysis of the interviews and self-assessment questionnaires taking into consideration such aspects as interest in the program and motivation to participate, their strengths and weaknesses identified by the participants, expectations of the program and future midwife-student collaboration, the program coordinator supported by a psychologist divided the participants into 12 mentorship pairs. The mentors and mentees first met at an introductory meeting and were informed that they could change their allocation to pairs, but none used that opportunity.

### Intervention

The clinical training was exclusively mentor-led. In addition to collaboration on the ward, there were four mentor-mentee meetings which served the purposes of establishing a successful mentorship relationship, setting training objectives, planning future activities and on-going assessment of the theoretical knowledge and clinical skills acquired by the mentee. The mentor evaluated progress made by the mentee and motivated her/him to continue their efforts. At the end of the clinical placement each student received a final appraisal describing their present strengths and areas for improvement. During the placement, each student could have individual consultations with the program coordinator who was an academic tutor.

### Data collection

In June 2019, at the end of the study when all participants had completed their clinical training, a focus group interview was conducted with all 12 mentees. Each student participated in the discussion and expressed their opinions and comments.

A focus group interview was selected because it is the main tool of qualitative research to identify what the participants’ perceptions and experiences are and to use the data to research a specific problem [31]. One of the strengths of the method is its inherent interaction between the participants giving insight into their reflections as a group and bringing together a variety of individual experiences and opinions. The moderator promotes debate, but, by definition, remains neutral, neither takes a position nor valuates the participants’ opinions. Data are collected in an unstructured manner. To prepare for a focus group, it is necessary to create the script for the moderator to use so that all topics of interest to the researcher(s) are included. The script, however, is not a list of specific questions for the moderator to ask expecting answers from each participant but a list of themes to guide an interactive discussion [31, 32].

The moderator was not a member of the staff who previously assessed the clinical skills performance by the participating students. The researchers were academic tutors responsible for the design of the mentoring program.

The session length was approximately 60 min. The discussion (participants’ responses) was recorded and transcribed verbatim. The quotes in this paper were translated from the original transcript in Polish into the English language for the purposes of this publication.

### Data analyses

The data (themes) from the transcribed interviews were analyzed by the researchers using the principles of thematic analysis in qualitative studies [33] and they identified five key questions to guide the focus group discussion (Table 1).

**Table 1:** Key questions guiding the focus group discussion on the clinical training

1. Did you get any valuable learning experience during your clinical training?	
2. How did this clinical training differ from your previous clinical training?	
3. What was your experience of one-on-one mentoring?	
4. Did the mentoring program meet your expectations?	
5. What do you think could be changed to make the proposed mentor-led clinical training more effective?	

In the next stage, all participants were asked to read the transcripts and identified themes to confirm the findings. The study findings were accepted and approved by all study participants.

The data were analyzed by two independent researchers and encoded (Student 1, Student 2, etc.). Next, four themes were identified and named: differences between traditional clinical training and a mentoring program; advantages and disadvantages of mentoring in clinical training of midwifery students; student-mentor relationship; and mentoring as an innovative and promising approach to clinical training for midwifery students. Below are quotes from students’ comments referring to each of the themes.

## Results

### Differences between traditional clinical training and a mentoring program

According to students, there were considerable differences between mentor-led clinical training and a traditional clinical placement. Currently, degree courses in midwifery in Poland offer clinical training in groups of 4 up to as many as 8 students supervised by one academic/clinical tutor. Mentoring, on the other hand, offers individualized clinical instruction involving the collaboration of two practitioners, an experienced midwife (mentor) and a student midwife (mentee).

The main difference between a traditional clinical placement and a mentoring program is in the number of students supervised by a midwife. I had individual training supervised by an assigned mentor, a one-on-one instruction. (Student 1)

Students emphasized that mentoring allowed them to do more:

I could perform the same activities several times which helped me to improve my clinical skills. (Student 4).

During a traditional placement usually we have no chance of working with the same midwife. Now, I had my own mentor and did everything just with her. (Student 7).

Clinical instruction in groups does not seem to fully satisfy the criteria of effective clinical skills training and the students’ expectations.

During a traditional clinical placement, usually in a group of 4-6 students, we often didn’t have much to do, because there was not enough work for all of us. (Student 5).

During a traditional clinical placement, with a relatively large group of students, it may be difficult for midwifery tutors to find enough tasks to occupy all students and allow them to show what clinical skills they have already mastered, but with a mentoring program students do not feel the pressure to compete with their colleagues for a chance to care for a patient and demonstrate their midwifery skills. The mentor ensures that the mentee uses her time well, involves her in all activities on the ward, shares her knowledge and creates opportunities to practice clinical skills.

There was not a single moment when I was just sitting there doing nothing. I was kept busy all the time. When there were no other tasks, I just sat down with the mentor to discuss clinical problems. (Student 10).

Students appreciated the opportunity to acquire and improve their clinical skills under the supervision of experienced mentors.

Very often I could plan some activity myself so my shift on the ward did not just consist of ‘obeying orders’ as it usually happens during a traditional clinical placement. Because I had my assigned mentor, I was able to plan and practice patient care under her supervision. (Student 3).

One student observed that in her case other members of the therapeutic team approved of mentoring:

It seemed to me that the clinical staff responded better to the presence of one student with one mentor than to several students supervised by just one tutor. (Student 10).

### Advantages and disadvantage of mentoring in clinical training of midwifery students

Mentoring offers students an opportunity of reliable assessment of their actual clinical skills and feedback provided by a mentor identifies the strengths and weaknesses of the performance or areas for improvement.

Of course during my earlier clinical training I acquired clinical skills, but with mentoring, I could show to an experienced midwife how I do things and she would tell me if I did right. (Student 2).

In students’ opinion mentoring considerably enhanced the quality of the clinical training they received which they hoped would be reflected in the quality of clinical care they would deliver.

In my opinion it’s a very effective method of instruction. An ideal method of clinical training as it develops your competencies as a midwife and allows you to actually perform some clinical tasks yourself and so it prepares you for your future career. (Student 4).

Students agreed that the effective use of midwifery skills and making informed decisions was possible in a safe and supportive learning environment.

I felt cared for and needed, I was treated as a member of the therapeutic team and that is why I never felt stressed and was more confident. (Student 9).

The students said that collaborative working with one assigned mentor led to a comprehensive, objective and fair appraisal of their skills.

Each student learns at her own pace. Learning in a system where one student learns under supervision of one clinical tutor allows the student to acquire necessary knowledge at her own pace which facilitates the integration of theory and practice. (Student 3).

I felt that working all the time with the same mentor I’ll be fairly assessed. (Student 8).

However, the participants observed that there were not enough mentors. One student pointed out that as mentor-led clinical training is not available to all, those who were not able to participate in the mentoring program, might feel they had not been given access to a better form of learning.

This is seems unfair to those students who could not participate in the program and a get a chance of experiencing what mentoring is. (Student 12).

### The student-mentor relationship

The concept of mentoring in clinical training of midwifery students involves collaborative working of midwives and students. If a midwifery mentor treats a student midwife as a member of the therapeutic team, it increases the student’s sense of belonging to the hospital department.

I first met my assigned mentor before the clinical placement started which positively influenced my attitude and our relation. (Student 1).

In students’ opinion good relations with other health care professionals help them to avoid mechanical memorizing of procedures and encourage critical thinking.

My mentor was always ready to answer all my questions. She explained the clinical problems I found difficult and encouraged me to perform some tasks independently. (Student 4).

Students valued the relationship between a student midwife and an experienced practitioner who provided support and counselling and shared knowledge.

My mentor proved to be friendly and caring. I could always ask her about anything I was not quite clear about. During the clinical placement the mentor wanted to share with me her knowledge and I could practice any particular skill with her. (Student 1).

The mentor was focused on sharing her knowledge and giving instruction just to me, which greatly increased the number and range of tasks I could perform. (Student 6).

Students emphasized how important it was to make clinical training a comfortable experience.

I didn’t feel any pressure. It was all very friendly. I could talk to my mentor about any topic, we often joked. We really felt like partners. (Student 5).

Students saw and appreciated their mentors’ involvement:

My mentor supported me during the performance of tasks and readily shared her knowledge. (Student 11).

Students realized that their mentors wanted to develop in them the ability of critical thinking and making informed decisions.

My mentor worked just with me, not a larger number of students, she knew very well what tasks I could perform myself without being supported or supervised. (Student 2).

Students felt that the mentoring relationship was based on mutual trust.

I think there was a greater deal of trust. I felt my mentor genuinely wanted to help me, not just critically assess my performance. (Student 9).

### Mentoring as an innovative and promising approach to clinical training for midwifery students

Students confirmed that innovative forms of clinical instruction improved the quality of their training.

With individualized instruction, it was easier for me to learn and get new skills. I was being prepared for emergency situations so that I knew what to do and what my role was. (Student 2).

They also appreciated a closer, less formal relationship between the mentor and mentee.

This individual approach and friendly attitude were very important, because I felt there’s someone who really cared about my education. (Student 6).

There was a general wish that all practical training would be mentor-led or that each midwifery student could have at least one chance of such individual mentoring.

Would it be possible for every midwifery student to have at least one opportunity of such individual clinical training during the 3-year course of study? (Student 12).

Students also perceived the potential effect of mentor-led clinical training on their future career. They thought newly qualified midwives would be better prepared to deliver clinical care, aware of their knowledge and skills and more confident.

My mentor showed me my strengths and weaknesses, thanks to the feedback she gave me I knew what I could do well and what needs further practice. My mentor set goals which I had to achieve and I learned a lot. (Student 5).

However, students observed that because mentoring was not a common approach to clinical training, some health care professionals did not fully understand the concept which was new for them.

Mentors need more recognition of their role, to avoid such comments by other midwives who are not mentors as ‘How is it that you are a mentor?’, ‘Who needs it?’, ‘Is that some special student?’ ‘Why just one student, not a group? (Student 3).

The participants saw that there were not enough mentors.

There is lack of mentors and my mentor often said that she felt the mentor’s role was somehow underestimated and there were not enough training opportunities for mentors. (Student 1).

They suggested that offering special training or guidance to hospital-based midwives could increase the number of midwives interested in mentoring for midwifery students.

## Discussion

The present system of midwifery education should be considerably revised to adapt clinical training to the changes in the health care system and the expectations of healthcare providers, healthcare users and student midwives themselves concerning the quality and value of pre-practice training. The findings of the study here reported demonstrate that traditional clinical placements do not satisfy all criteria of effective acquisition of new knowledge and clinical skills by midwifery students to prepare them for clinical practice.

University schools offering degree courses in midwifery should seek novel solutions to improve the quality of training. For instance, simulation, low- and high-fidelity, cannot replace real-world patient care. To effectively prepare a midwifery student for clinical practice, all clinical procedures must be ultimately practiced on the ward where students are involved in actual patient care.

McKenna shows that access to mentoring in midwifery may be a key element to improve the quality of clinical training [16].

In many countries mentoring as a strategy of providing support in the acquisition of new knowledge has become an integral part of professional education [28, 34]. The present study confirms that mentoring perfectly meets the actual needs of student midwives. Having a dedicated and formally assigned mentor helps students to find their place in the new hospital setting and become more confident during their clinical placement. Monitoring could be adopted not only in clinical training at undergraduate level but also in continuing education of newly graduated midwives employed in healthcare institutions [10, 28, 35, 36]. Mentoring which involves a long-term professional relationship [37] may be implemented in a workplace.

The present study focused on the experiences of midwifery students who completed their first ever cycle of clinical mentoring. As midwifery educators we wanted to know what they thought of clinical mentoring as an innovative method of instruction and whether in their opinion it deserved its place in the future midwifery curricula. Obviously, equally important is the experience of mentors and their views of the strengths and weaknesses of clinical mentoring. This aspect of mentorship definitely needs further studies.

In our study, students appreciated the mentors’ efforts and their involvement and thought that the most valuable benefits of mentoring included a personal relationship with the mentors and their help in identifying the roles and responsibilities of a midwife, counselling, developing clinical skills and enhancing evidence-based practical knowledge. As shown by other authors mentoring has a key role in providing a safe learning environment and introducing mentees to the real world of clinical practice [10, 23, 28, 30, 38]. According to the participants of the study here reported, a friendly atmosphere and good relations with the clinical mentor are essential for the effective acquisition of new knowledge and clinical skills. As previously shown by Davis et al., mutual trust and respect between the mentor and mentee are important for achieving the goals of mentoring [28, 39]. We demonstrated that the trust and support of mentors boosted students self-confidence.

According to the midwives acting as mentors in the present program, this personal relationship between mentor and mentee increases the effectiveness of instruction and facilitates reaching of its objectives as instruction can be tailored to the needs and actual clinical competence of individual students. In a Swedish study, Jansson et al. show that when an instructor does not have full knowledge of students’ clinical competence this may lead to situations when trainees may undertake patient care tasks for which they are unprepared and actually harm patients. Mentoring relationships built on trust and mutual accountability and responsibility create opportunities for a supportive clinical learning environment, enhance acquisition of clinical skills and boost students’ self-confidence.

Snowden & Hardy suggest that psychological stress and fear of failure may interfere with normal learning process [40] Mentors are seen as those who can develop in mentees confidence about their professional future and faith in their abilities. A mentee should feel comfortable in her/his relation with a mentor [30, 41]. Making a student part of the team, interest in the student and her/his role, showing them what the patients need and how to deliver care, increasing students’ confidence about their knowledge and competencies were listed by students as the most important benefits of mentoring.

As in other studies [30, 42], students perceived working collaboratively with a midwife as an excellent opportunity to acquire new knowledge and practice skills under the supervision of an experienced practitioner. By helping to achieve the training goals and identifying what should be improved, mentoring was seen by students as a superior way of preparing them for real-world clinical practice.

Whatever its merits, mentoring has not been yet commonly adopted in the training of midwifery students in Poland. The barriers include cost, time limits, and the lack of clear rules governing mentoring as an approach to teaching of clinical skills and its formal recognition [10]. In our study, participants observed that mentoring is not commonly accepted in clinical training which leads to a number of problems. The lack of standards, limited knowledge of the principles of mentoring and how to become a mentor, and frequently lack of recognition of the mentor’s role are responsible for the shortage of mentors [7, 43]. It is necessary to increase the awareness of the advantages of mentoring among the clinical staff and to offer professional help to those who want to act as mentors.

In this study, mentors also expressed their doubts concerning wider adoption of mentoring in the midwifery curricula. Some believed that introduction of training courses for midwives working in hospitals could increase the number of mentors and general interest in mentor-led clinical teaching.

Peer mentoring as proposed by McKellar et al. could be another solution. The success of their midwifery student peer mentoring program suggests that it may be implemented in other educational institutions [9]. Involvement of more experienced third-year midwifery students in mentoring first-year students could be a strategy of providing support during clinical placements.

Clinical mentoring program for midwifery students should be implemented in all medical universities offering degree courses in midwifery and accessible to all midwifery students [6, 44]. Mentoring is reciprocally beneficial to the mentor and mentee [9]. It gives satisfaction to both parties, improves interpersonal relations, reduces stress levels and helps to improve career opportunities [34, 42]. Mentoring is a training system widely used with different professional groups and by different institutions. This important initiative should be implemented and developed within a variety of educational and research programs at institutions responsible for quality midwifery education, irrespective of the country [45].

We present the first Polish study of the experiences and opinions of midwifery students who completed a mentor-led clinical training. Although the study group was small, the analysis offered a review of midwifery students’ learning experiences. A limitation of the study is the fact that this particular mentoring program was implemented in just one hospital in one city while each student could train under the guidance of her mentor for just 3 weeks. It may not reflect the perceptions of other students from other Polish medical universities concerning their clinical training. Importantly, members of the focus group were students of the second year and third, final year of the Bachelor course in midwifery and their clinical skills and competencies were obviously at much higher level than those of first-year students. That experience might have contributed to their good collaboration with mentors and a very positive opinion of mentoring. Further studies would be needed to find out how first-year students perceive mentoring. What would they expect to gain from mentoring? How would they collaborate with mentors? Another interesting area of research would be learning more about the opinions of mentors and comparing them with the opinions of students to find out the expectations and perceptions of mentoring in each of these groups and their actual experiences of mentoring.

## Conclusions

The evaluation we performed allows the conclusions that the relationship between an experienced midwife as mentor and a less experienced midwifery student may contribute to the development in the latter of professional competencies, help to introduce her/him to clinical practice in a real-world hospital setting and facilitate personal development. The use of innovative methods of instruction enhances the quality of clinical training which should translate into high-quality patient care in the future. Mentorship is reciprocally beneficial, but it should focus on improving the effectiveness of the junior partner’s performance.

Mentoring in midwifery has a great potential and may contribute to developing an entirely new model of education and training with more effective acquisition and retention of new knowledge and clinical skills. The present system of midwifery education in Poland needs considerable revision and clinical training has to be changed to bring its level up to the international standards and satisfy the needs and expectations of health care professionals, educators, students and healthcare recipients. A traditional model of clinical teaching/learning based on tight schedules and group instruction does not contribute to efficient teaching/learning of a wide range of midwifery skills.

## Supplementary Information

**Additional file 1:.** Mentors Bio-data Questionnaire

**Additional file 2:.** Student Bio-data Questionnaire

## Data Availability

All data generated or analyzed during this study are included in this published article.

## References

[1] Programme for complex protection of reproductive health in Poland 2016–2020. Journal of Laws, 2018, item 1510. file:///C:/Users/malgo/AppData/Local/Temp/Program_kompleksowej_ochrony_zdrowia_prokreacyjnego_w_Polsce_w_latach_2016-2020_-_aktualizacja_2019_r_-_wersja_do_powieszenia_na_stronie.pdf.

[2] Regulation of Minister of Health of 16 August 2018 on the organizational standards of perinatal care. Journal of Laws, item 1756. https://isap.sejm.gov.pl/isap.nsf/DocDetails.xsp?id=WDU20180001756.

[3] Royal College of Midwives (2014). High quality midwifery care.

[4] Stefaniak M (2019). Good practices in midwifery - mentoring in practical education of students. Położna Nauka i Praktyka.

[5] Commission Regulation (EU) No 623/2012 of 11 July 2012 amending Annex II to Directive 2005/36/EC of the European Parliament and of the Council on the recognition of professional qualifications Text with EEA relevance. Off J Eur Union. 2012.

[6] Dobrowolska B, McGonagle I, Kane R, Jackson CS, Kegl B, Bergin M, Cabrera E, Cooney-Miner D, Di Cara V, Dimoski Z, Kekus D, Pajnkihar M, Prlić N, Sigurdardottir AK, Wells J, Palese A (2016). Patterns of clinical mentorship in undergraduate nurse education: a comparative case analysis of eleven EU and non-EU countries. Nurse Educ Today.

[7] Gibson T, Heartfield M (2005). Mentoring for nurses in general practice: an Australian study. J Interprof Care.

[8] Harvey S, Uren CD (2019). Collaborative learning: application of the mentorship model for adult nursing students in the acute placement setting. Nurse Educ Today.

[9] McKellar L, Kempster C (2017). We're all in this together: midwifery student peer mentoring. Nurse Educ Pract.

[10] Lennox S, Skinner J, Foureur M (2008). Mentorship preceptorship and clinical supervisio: three key processes for supporting midwives. N Z Coll Midwives J.

[11] Pendleton M. Student Mentoring and Peer Tutoring: a Literature Review. RMIT University viewed 10 November 2015.

[12] Edgecombe K, Wotton K, Gonda J, Mason P (1999). Dedicated education units: 1. A new concept for clinical teaching and learning. Contemp Nurse.

[13] Wilczek-Rużycka E, Majda A, Walewska E, Kózka M, Czaja , Gajos, M. 2004 Preparation of mentor nurses to conduct practical classes with nursing students: a package of training materials. , Jagiellonian University Publishing House. Cracow.

[14] Morton-Cooper A, Palmer A (1993). Mentoring and preceptorship: a guide to support roles in clinical practice.

[15] White R, Ewan C (1991). Clinical teaching in nursing.

[16] McKenna L (2003). Nurturing the future of midwifery through mentoring. Aust J Midwifery.

[17] Waters D, Clark M, Harris-Ingall A, Dean-Jones M (2003). Evaluation of a pilot mentoring programme for nurse managers. J Adv Nurs.

[18] Lobo C, Arthur A, Lattimer V. Collaborative learning in practice (CLiP) for preregistration nursing students. University of East England; 2014.

[19] Bradshaw C, Murphy Tighe S, Doody O (2018). Midwifery students’ experiences of their clinical internship: a qualitative descriptive study. Nurse Educ Today.

[20] Schulz PM, Dunne CL, Burdett-Jones D, Gamble NS, Kosiak MM, Neal JM, Baker GE (2018). Evaluation of strategies designed to enhance student engagement and success of indigenous midwifery students in an away-From-Base bachelor of midwifery program in Australia: a qualitative research study. Nurse Educ Today.

[21] Cummins AM, Denney-Wilson E, Homer CSE (2015). The experiences of new graduate midwives working in midwifery continuity of care models in Australia. Midwifery..

[22] McKenzie B (1995). Friends in high places: how to achieve your ambitions, goals and potential with the help of a mentor.

[23] Casey DC, Clark L (2011). Roles and responsibilities of the student nurse mentor: an update. Br J Nurs.

[24] Wilkes Z (2006). The student–mentor relationship: a review of the literature. Nurs Stand.

[25] Avis M, Mallik M, Fraser D (2013). “Practising under your own pin” - a description of the transition experiences of newly qualified midwives. J Nurs Manag.

[26] Skirton H, Stephen N, Doris F, Cooper M, Avis M, Fraser D (2012). Preparedness of newly qualified midwives to deliver clinical care: an evaluation of pre-registration midwifery education through an analysis of key events. Midwifery..

[27] Roman M (2001). Mentors, mentoring. Medsurg Nurs.

[28] Cummins AM, Denney-Wilson E, Homer CSE (2017). The mentoring experiences of new graduate midwives working in midwifery continuity of care models in Australia. Nurse Educ Pract.

[29] Canadian Nurses Association. Achieving excellence in profession practice: a guide to preceptorship and mentoring. Ottawa: Canadian Nurses Association; 2004. www.cna-aiic.ca.

[30] Warne T, Johansson UB, Papastavrou E, Tichelaar E, Tomietto M, Van den Bossche K, Saarikoski M (2010). An exploration of the clinical learning experience of nursing students in nine European countries. Nurse Educ Today.

[31] Morgan DL (1988). Focus group as qualitative research.

[32] Burnar P (1991). A method of analysing interview transcripts in qualitative research. Nurse Educ Today.

[33] Braun V, Clarke V (2006). Using thematic analysis in psychology. Qual Res Psychol.

[34] Fisher M, Webb C (2009). What do midwifery mentors need? Priorities and impact of experience and qualification. Learn Health Soc Care.

[35] Davies S, Mason J (2009). Preceptorship for newly-qualified midwives: time for a change. Br J Midwifery.

[36] Saultz JW (2003). Defining and measuring interpersonal continuity of care. Ann Fam Med.

[37] Hynes-Gay P, Swirsky H (2001). Mentorship in nursing. Registered Nurse.

[38] Claeys M, Deplaecie M, Vanderplancke T, Delbaere I, Myny D, Beeckman D, Verhaeghe S (2015). The difference in learning culture and learning performance between a traditional clinical placement, a dedicated education unit and work-based learning. Nurse Educ Today.

[39] Davis D, Foureur M, Clements V, Brodie P, Herbison P (2012). The self reported confidence of newly graduated midwives before and after their first year of practice in Sydney, Australia. Women Birth.

[40] Snowden M, Hardy T (2013). Peer mentorship and positive effects on student mentor and mentee retention and academic success. Widening Participation Lifelong Learn.

[41] Bower FL. Mentoring others. W: Bower F.L., ed. Nurses taking the lead. Personal qualities of effective leadership. Philadelphia: W.B. Saunders. 2010;255–275.

[42] Kensington M (2006). The faces of mentoring in New Zealand: realities for the new graduate midwife. N Z Coll Midwives J.

[43] Górka E, Kunecka D, Szylińska A, Ptak M (2019). Coaching and mentoring in nursing practice. Pomeranian J Life Sci.

[44] Moran M, Banks D (2016). An exploration of the value of the role of the mentor and mentoring in midwifery. Nurse Educ Today.

[45] Jansson I, Ene KW (2016). Nursing students’ evaluation of quality indicators during learning in clinical practice. Nurse Educ Pract.

