# Supplementary material for: Mentoring in the clinical training of midwifery students - a focus study of the experiences and opinions of midwifery students at the Medical University of Warsaw participating in a mentoring program
Source: BMC Med Educ. 2020 Oct 30;20:394. doi: 10.1186/s12909-020-02324-w (PMC7602316; doi:10.1186/s12909-020-02324-w)
Supplement: Supplementary file 1 — Additional file 1:. Mentors Bio-data Questionnaire [file 12909_2020_2324_MOESM1_ESM.docx]

**Mentors Bio-data Questionnaire**

Name and surname:__________________________________________________________

Seniority in the midwife's profession:____________________________________________

Place of work:______________________________________________________________

E-mail:______________________________________________

| 1. | Age: |
| --- | --- |
| 2. | How many years have you worked in this unit? |
| 3. | Position: |
| 5. | Experience before this clinical internship: |
| 6. | Description of experiences from previous internships with students: |
| 7. | Motivation to participate in the program "Good practices in midwifery - mentoring in practical education of students": |
| 8. | Expectations concerning participation in the program "Good Practices in Obstetrics - mentoring in practical education of students": |
