# Supplementary material for: Mentoring in the clinical training of midwifery students - a focus study of the experiences and opinions of midwifery students at the Medical University of Warsaw participating in a mentoring program
Source: BMC Med Educ. 2020 Oct 30;20:394. doi: 10.1186/s12909-020-02324-w (PMC7602316; doi:10.1186/s12909-020-02324-w)
Supplement: Supplementary file 2 — Additional file 2:. Student Bio-data Questionnaire [file 12909_2020_2324_MOESM2_ESM.docx]

**Student Bio-data Questionnaire**

Name and Surname:

Year of studies:

E-mail:

Clinical internship:

Unit ________________________ Date __________________________________________

| 1. | Age: |
| --- | --- |
| 2. | Preferred internship branch with justification: |
| 3. | Interests: |
| 4. | Average grade for the last completed year of studies: |
| 5. | Experience before this clinical internship: |
| 7. | Motivation to participate in the program "Good practices in midwifery - mentoring in practical education of students": |
| 8. | Images (expectations) concerning participation in the program "Good Practices in Obstetrics - mentoring in practical education of students": |
| 9. | Expectations (ideas) about the mentor: |
| 10. | Your strengths: |
| 11. | Your weaknesses: |
| 12. | What specific skills would you like to achieve during the class and what should they include?  What kind of help do you expect? |
